# Supplementary material for: Diagnostic and antibiotic use practices among COVID-19 and non-COVID-19 patients in the Indonesian National Referral Hospital
Source: PLoS One. 2024 Mar 7;19(3):e0297405. doi: 10.1371/journal.pone.0297405 (PMC10919621; doi:10.1371/journal.pone.0297405)
Supplement: S1 Fig — For this figure, antibiotics in the Watch category was divided to Watch and Watch+. Watch+ category comprises antibiotics in the Watch category with anti-MRSA activity (e.g. vancomycin) or antipseudomonal activity (e.g. antipseudomonal cephalosporin, antipseudomonal penicillin and carbapenems). (DOCX) [file pone.0297405.s001.docx]

**S1 Figure**. Proportional consumption of (A) parenteral and (B) oral antibiotics by AWaRe categorization among all admissions (n=91,960) of all inpatients (n=60,228) between 2019 and 2020


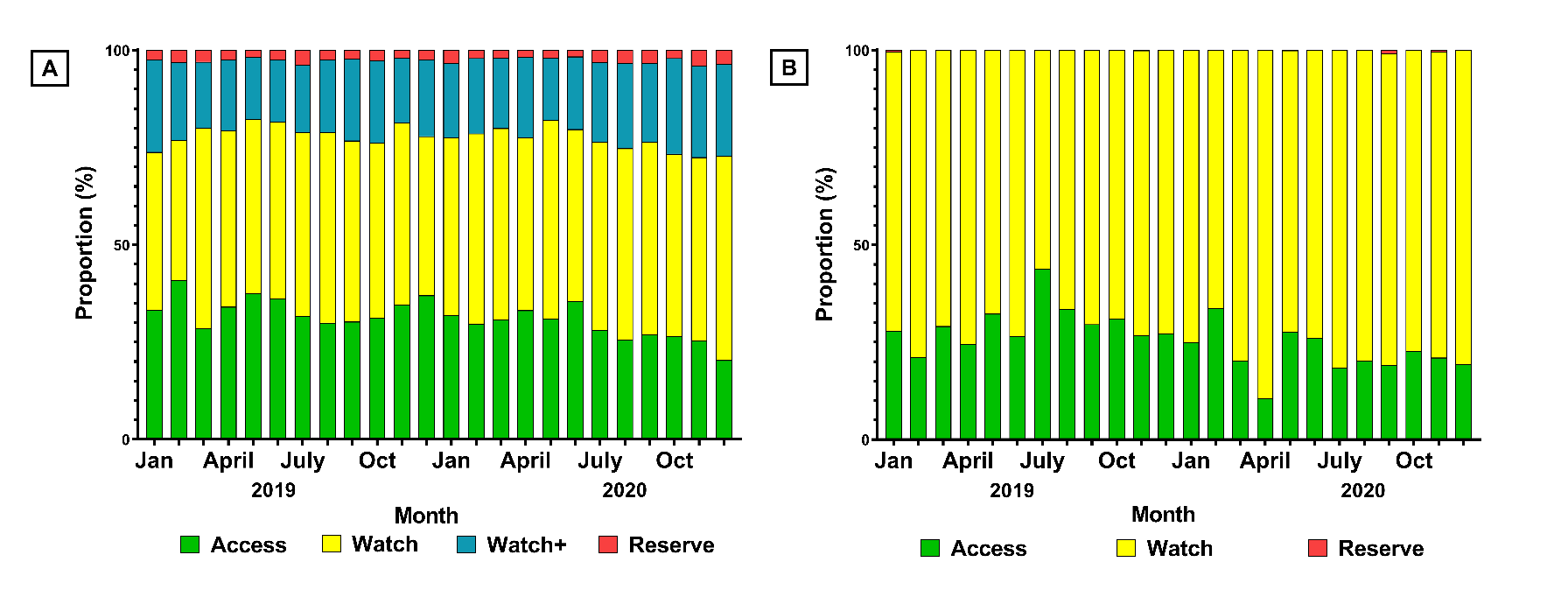


**Footnote:** For this figure, antibiotics in the Watch category was divided to Watch and Watch+. Watch+ category comprises antibiotics in the Watch category with anti-MRSA activity (e.g. vancomycin) or antipseudomonal activity (e.g. antipseudomonal cephalosporin, antipseudomonal penicillin and carbapenems).
