# Supplementary material for: Diagnostic and antibiotic use practices among COVID-19 and non-COVID-19 patients in the Indonesian National Referral Hospital
Source: PLoS One. 2024 Mar 7;19(3):e0297405. doi: 10.1371/journal.pone.0297405 (PMC10919621; doi:10.1371/journal.pone.0297405)
Supplement: S2 Fig — J01A: tetracyclines; J01B: amphenicols; J01C: beta-lactam antibacterials, penicillins; J01D: other beta-lactam antibacterials; J01E: sulfonamides and trimethoprim; J01F: macrolides, lincosamides and streptogramins; J01G: aminoglycoside antibacterials; J01M: quinolone antibacterials; J01R: combinations of antibacterials; J01X: other antibacterials, P01A: agents against amoebiasis and other protozoal diseases. (DOCX) [file pone.0297405.s002.docx]

**S2 Figure.** Consumption of parenteral antibiotics (DDD per 1,000 patient days) by pharmacological subgroups among all admissions (n=91,960) of all inpatients (n=60,228) between 2019 and 2020


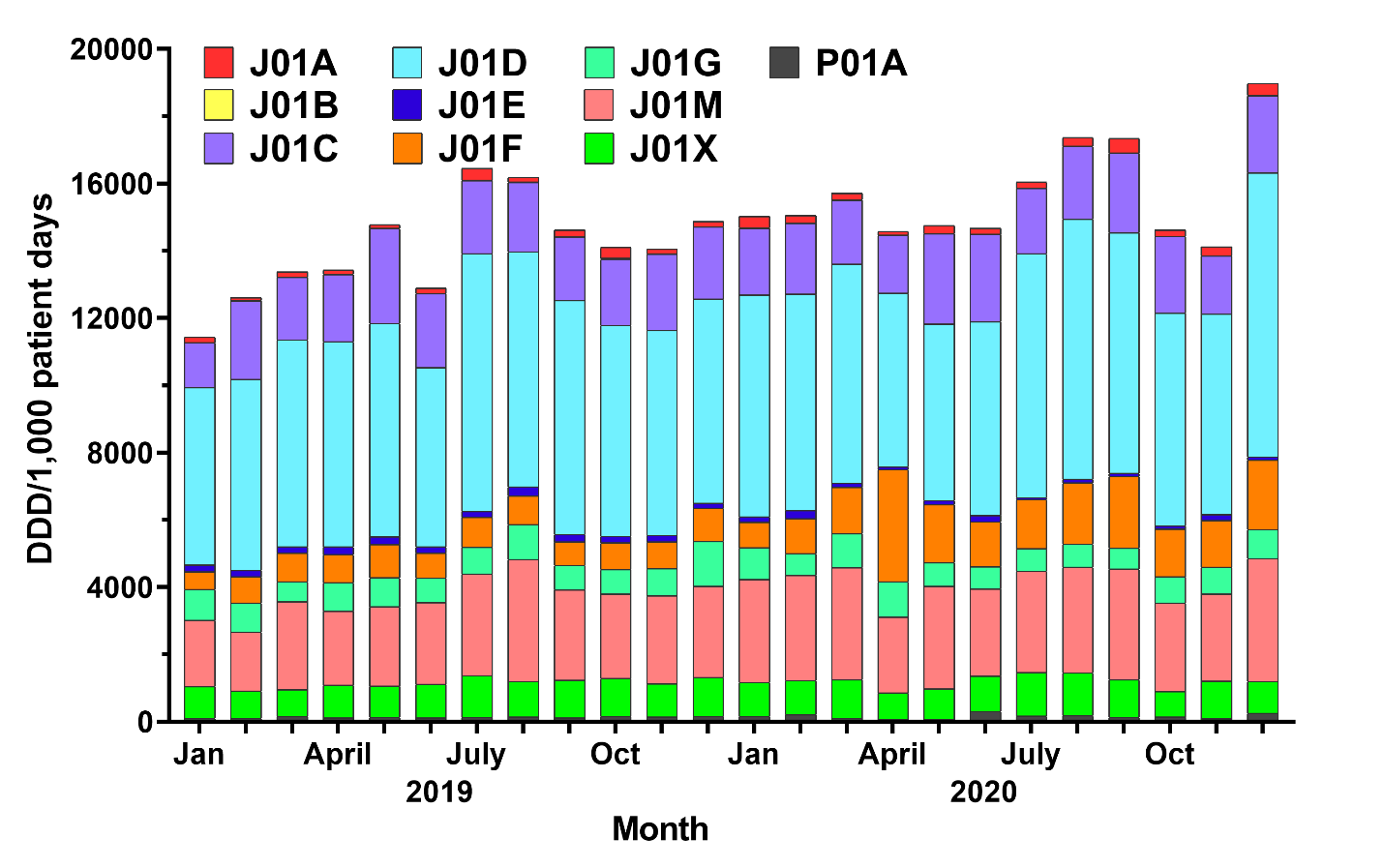


**Footnote:** J01A: tetracyclines; J01B: amphenicols; J01C: beta-lactam antibacterials, penicillins; J01D: other beta-lactam antibacterials; J01E: sulfonamides and trimethoprim; J01F: macrolides, lincosamides and streptogramins; J01G: aminoglycoside antibacterials; J01M: quinolone antibacterials; J01R: combinations of antibacterials; J01X: other antibacterials, P01A: agents against amoebiasis and other protozoal diseases
