# Supplementary material for: Diagnostic and antibiotic use practices among COVID-19 and non-COVID-19 patients in the Indonesian National Referral Hospital
Source: PLoS One. 2024 Mar 7;19(3):e0297405. doi: 10.1371/journal.pone.0297405 (PMC10919621; doi:10.1371/journal.pone.0297405)
Supplement: S3 Fig — Parenteral antibiotics being prescribed within the first calendar day that a parenteral antibiotic was started were regarded as initial parenteral antibiotics. Patients who received a parenteral antibiotic for at least four consecutive days was used as a surrogate for severe infection, with the first calendar equal to the start date of parenteral antibiotics. Patients who died, were discharged to a hospice or transferred to other hospital before completing four consecutive days of parenteral antibiotics and had parenteral antibiotics continuously until the day prior to death, hospice discharge or transfer were also included as patients with severe infection. (DOCX) [file pone.0297405.s003.docx]

**S3 Figure**. Distribution of initial antibiotic among 19,170 patients with severe infection


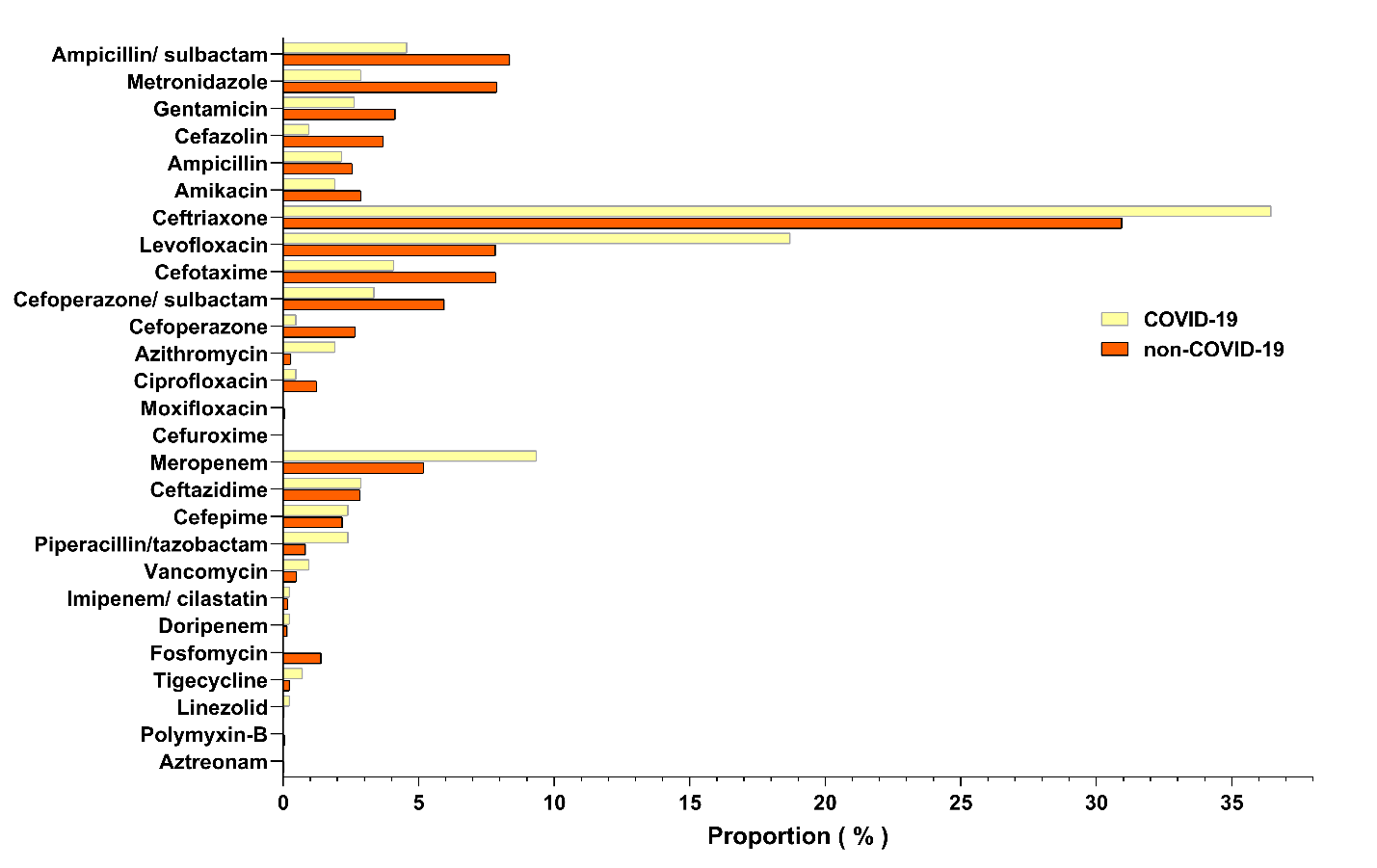

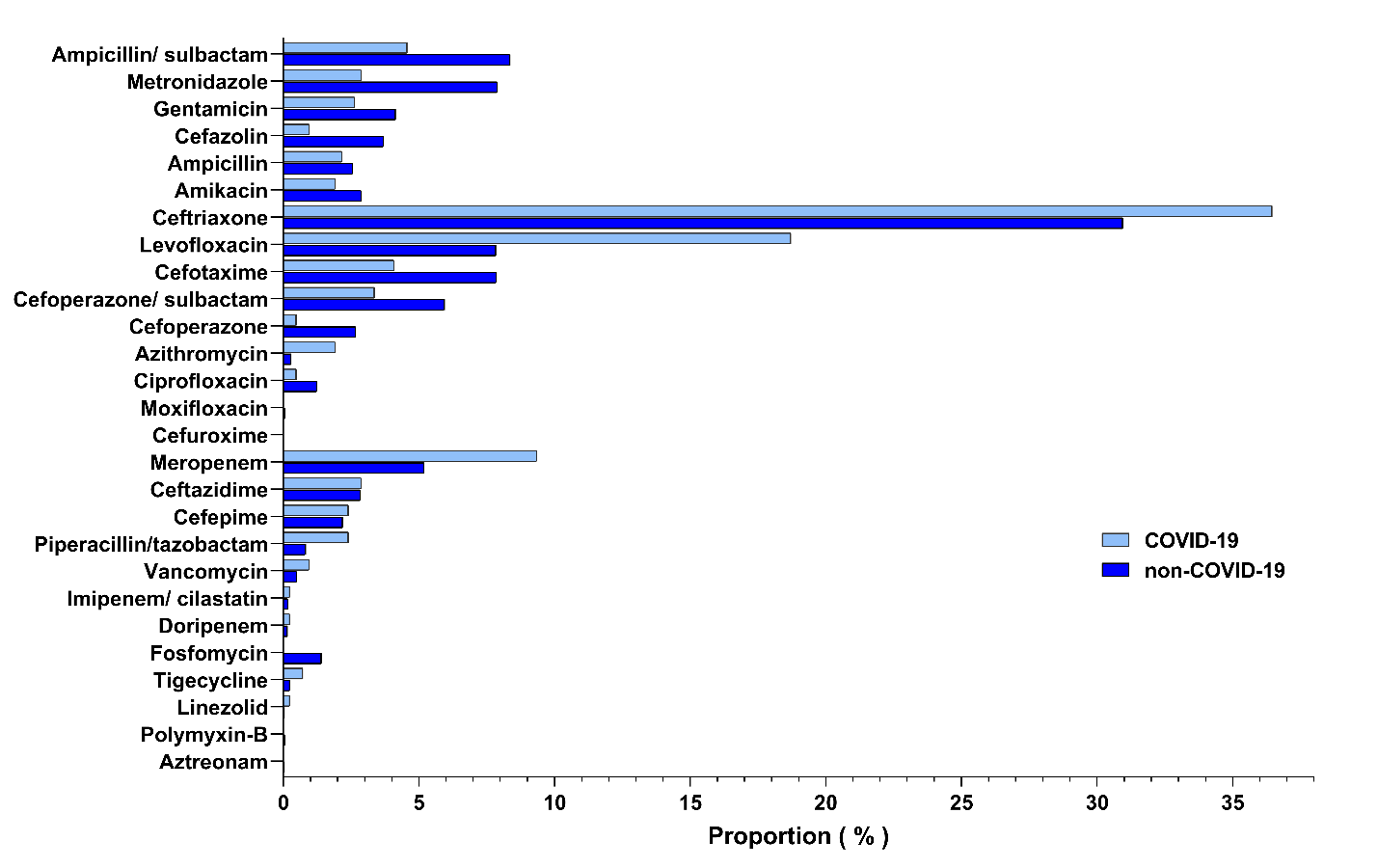

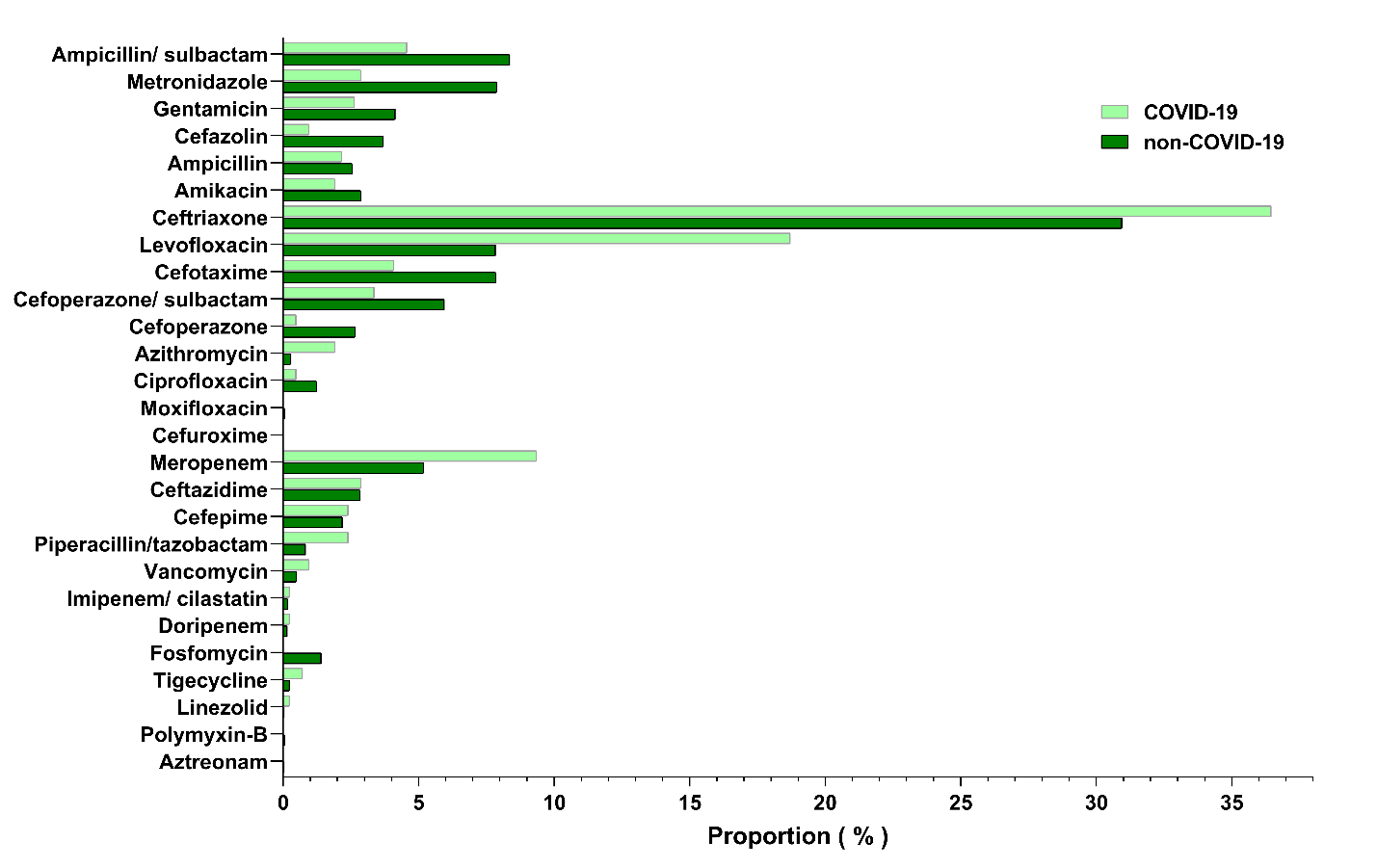

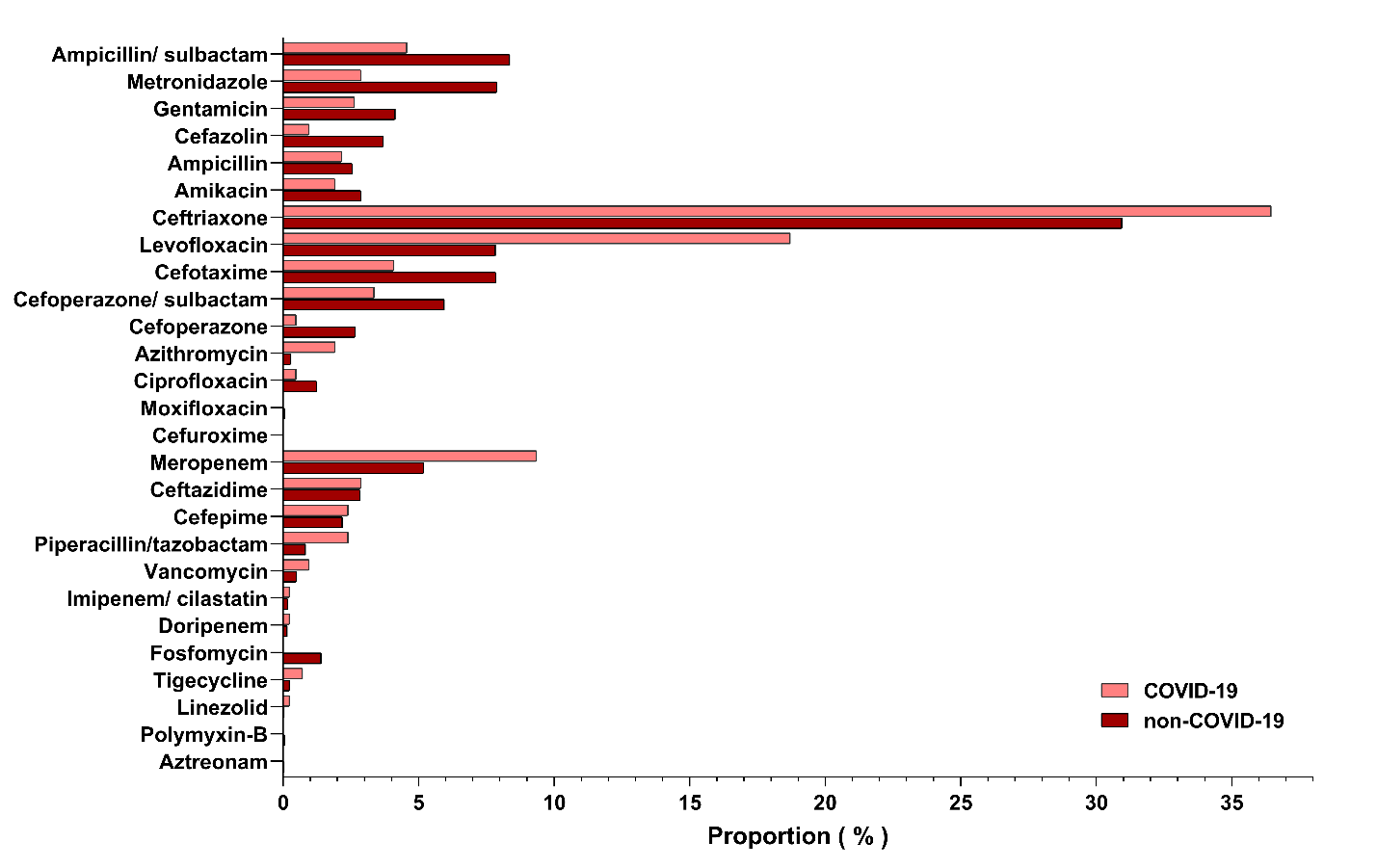


**Footnote:** Parenteral antibiotics being prescribed within the first calendar day that a parenteral antibiotic was started were regarded as initial parenteral antibiotics. Patients who received a parenteral antibiotic for at least four consecutive days was used as a surrogate for severe infection, with the first calendar equal to the start date of parenteral antibiotics. Patients who died, were discharged to a hospice or transferred to other hospital before completing four consecutive days of parenteral antibiotics and had parenteral antibiotics continuously until the day prior to death, hospice discharge or transfer were also included as patients with severe infection.
