# Supplementary material for: Diagnostic and antibiotic use practices among COVID-19 and non-COVID-19 patients in the Indonesian National Referral Hospital
Source: PLoS One. 2024 Mar 7;19(3):e0297405. doi: 10.1371/journal.pone.0297405 (PMC10919621; doi:10.1371/journal.pone.0297405)
Supplement: S4 Fig — Cumulative incidence of (A) mortality, (B) having blood culture sampled, and (C) discontinuation of parenteral antibiotics among 19,170 patients with severe infection. (DOCX) [file pone.0297405.s004.docx]

**
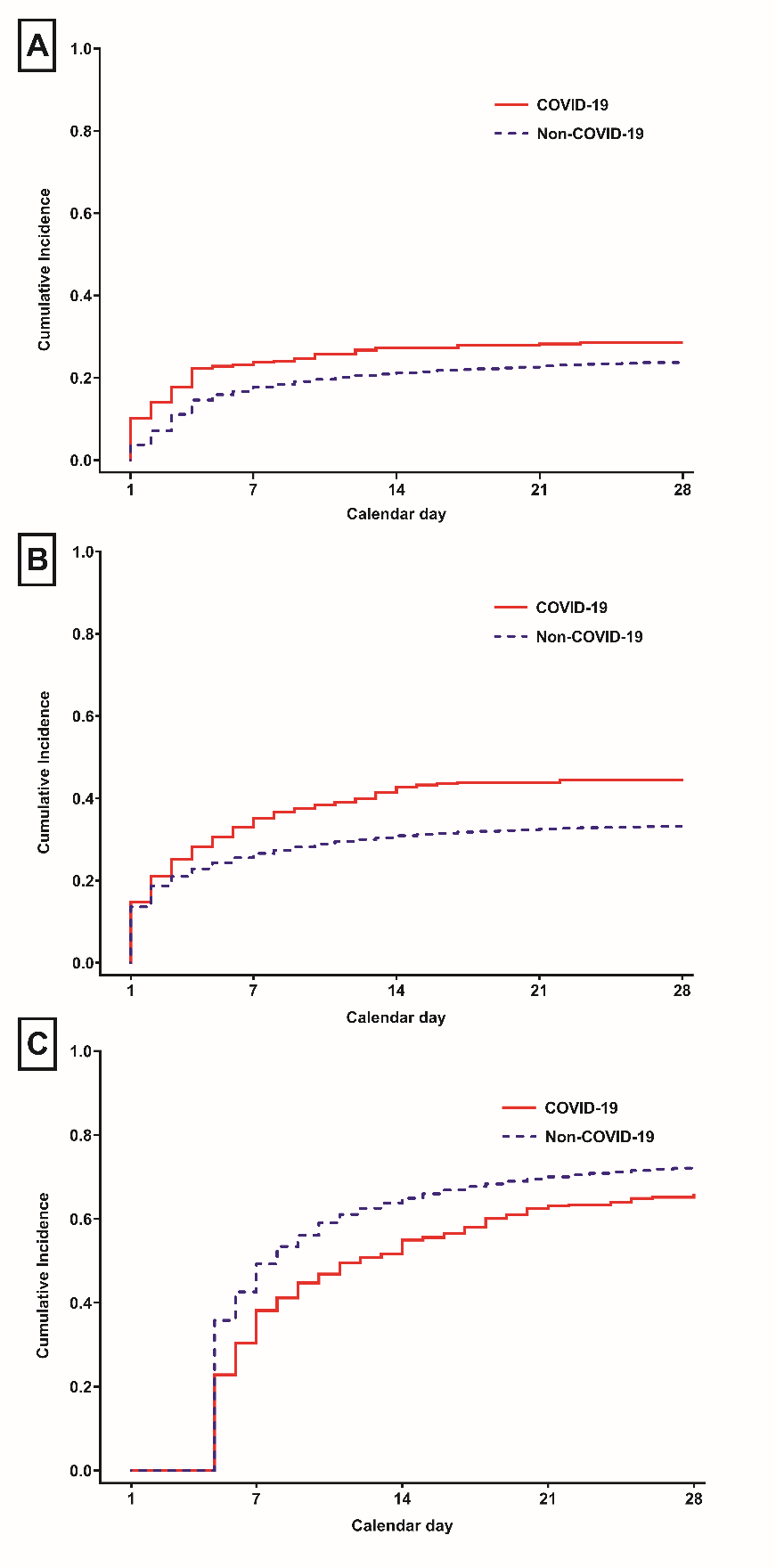
S4 Figure.** Cumulative incidence of (A) mortality, (B) having blood culture sampled, and (C) discontinuation of parenteral antibiotics among 19,170 patients with severe infection
