# Supplementary material for: Diagnostic and antibiotic use practices among COVID-19 and non-COVID-19 patients in the Indonesian National Referral Hospital
Source: PLoS One. 2024 Mar 7;19(3):e0297405. doi: 10.1371/journal.pone.0297405 (PMC10919621; doi:10.1371/journal.pone.0297405)
Supplement: S5 Fig — (DOCX) [file pone.0297405.s005.docx]

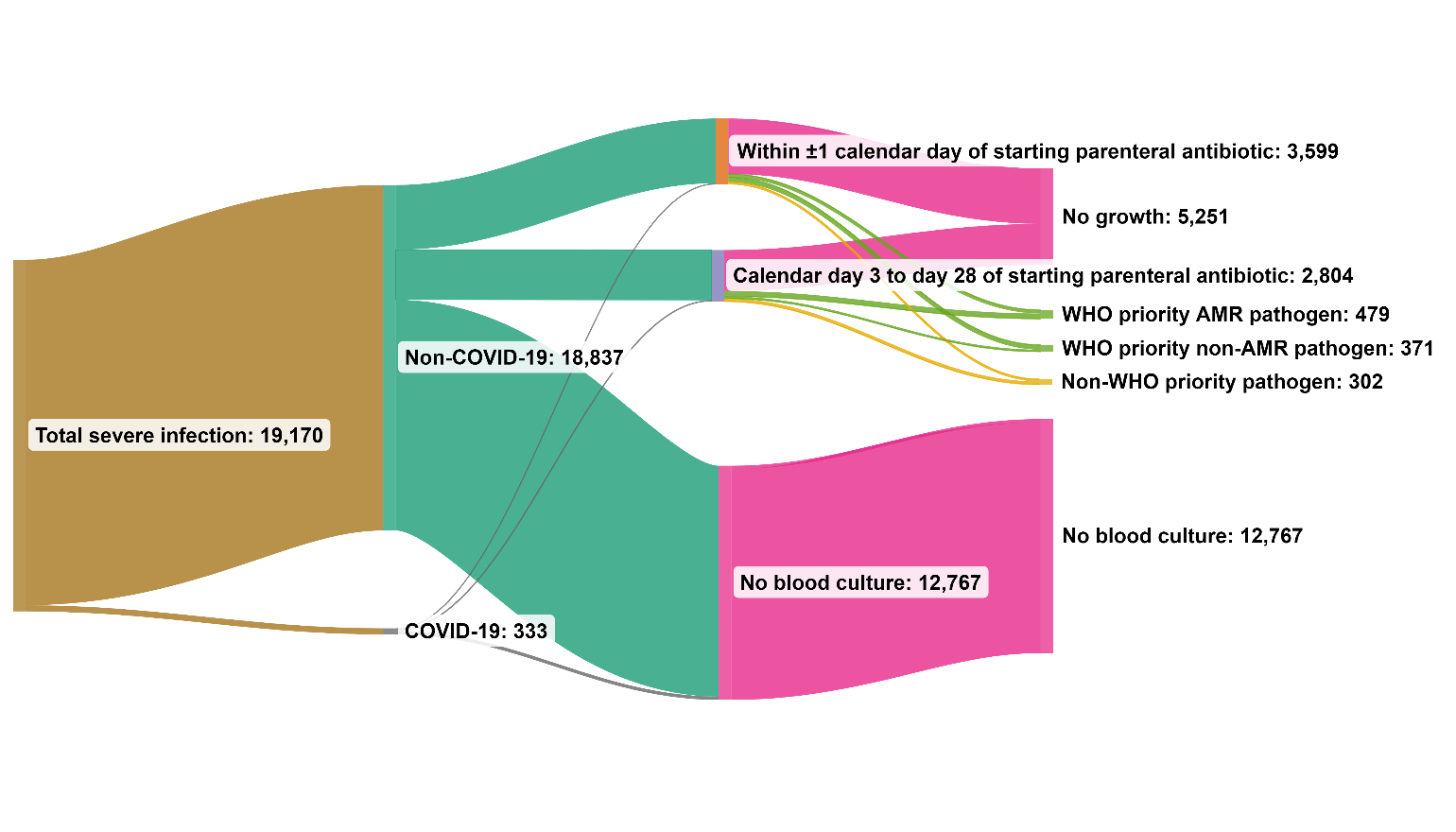
**S5 Figure.** Sankey diagram showing first blood culture test and blood culture result among 19,170 patients with severe infection
