# Supplementary material for: Diagnostic and antibiotic use practices among COVID-19 and non-COVID-19 patients in the Indonesian National Referral Hospital
Source: PLoS One. 2024 Mar 7;19(3):e0297405. doi: 10.1371/journal.pone.0297405 (PMC10919621; doi:10.1371/journal.pone.0297405)
Supplement: S6 Fig — For this figure, antibiotics in the Watch category was divided to Watch and Watch+. Watch+ category comprises antibiotics in Watch category with anti-MRSA activity (e.g. vancomycin) or antipseudomonal activity (e.g. antipseudomonal cephalosporin, antipseudomonal penicillin and carbapenems. (DOCX) [file pone.0297405.s006.docx]

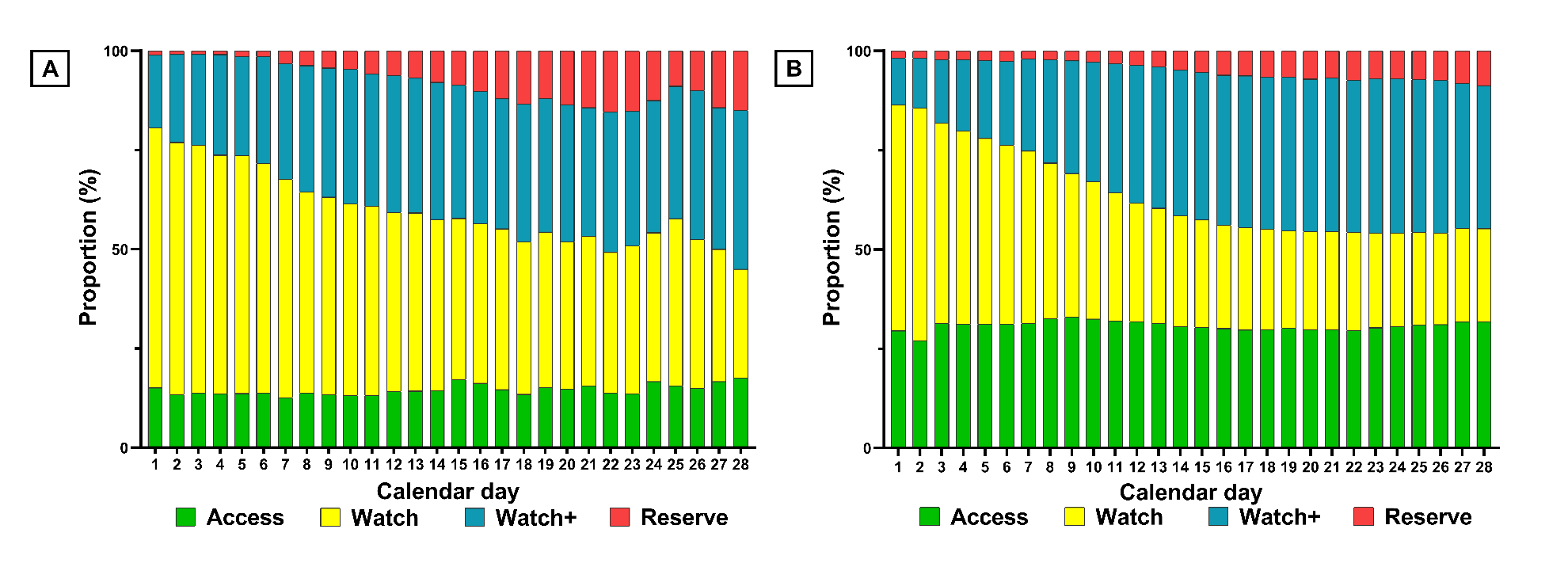
**S6 Figure.** Proportional consumption of parenteral antibiotics given to (A) 333 COVID-19 patients and (B) 18,837 non-COVID-19 patients with severe infection by AWaRe categorization over 28 calendar days

**Footnote:** For this figure, antibiotics in the Watch category was divided to Watch and Watch+. Watch+ category comprises antibiotics in Watch category with anti-MRSA activity (e.g. vancomycin) or antipseudomonal activity (e.g. antipseudomonal cephalosporin, antipseudomonal penicillin and carbapenems.
