# Supplementary material for: Diagnostic and antibiotic use practices among COVID-19 and non-COVID-19 patients in the Indonesian National Referral Hospital
Source: PLoS One. 2024 Mar 7;19(3):e0297405. doi: 10.1371/journal.pone.0297405 (PMC10919621; doi:10.1371/journal.pone.0297405)
Supplement: S1 Table — (DOCX) [file pone.0297405.s007.docx]

**S1 Table**. Baseline characteristics of all patients hospitalized at the Indonesian national referral hospital, Jakarta, Indonesia from 1 January 2019 to 31 December 2020.

| **Parameters** | **Total** | **COVID-19** | **Non-COVID-19** | **P values** |
| --- | --- | --- | --- | --- |
| Total number of admissions | 91,960 | 1,373 | 90,587 |  |
| Total number of inpatients (de-duplicated) | 60,228 | 1,311 | 58,917 |  |
| Number of patient-days | 583,248 | 11,541 | 571,707 |  |
| Median age (years old, IQR)* | 39 (17-55) | 41 (26-57) | 39 (17-55) | 0.47 |
| Sex* |  |  |  |  |
| Female | 32,167 (53.4%) | 646 (49.5%) | 31,521 (53.5%) | 0.002 |
| Male | 28,061 (46.6%) | 665 (50.7%) | 27,396 (46.5%) |  |
| Payor** |  |  |  |  |
| Universal health coverage | 77,149 (83.9%) | 1,373 (100%) | 75,776 (83.6%) | <0.001 |
| Private insurance | 2,071 (2.3%) | - | 2,071 (2.3%) |  |
| Self-paid | 12,740 (13.8%) | - | 12,740 (7.59%) |  |
| Number of admissions with at least one dose of parenteral antibiotic** | 35,766 | 599 | 35,167 |  |
| Proportion of admissions with at least one dose of parenteral antibiotic** | 38.9%  (35,766/91,960) | 43.6% (599/1,373) | 38.8%  (35,167/90,587) | <0.001 |
| Number of admissions with severe infection*** | 23,317 | 336 | 22,981 |  |
| Number of patients with severe infection*** | 19,170 | 333 | 18,837 |  |
| Proportion of patients with severe infection*** | 31.8%  (19,170/60,288) | 25.4% (333/1,311) | 31.9%  (18,837/58,917) | <0.001 |
| Total antibiotic consumption (DDD per 1,000 patient-days) | 624.1 | 1,502.8 | 606.3 |  |
| Access category (%) | 182.4 (29.3%) | 82.2 (5.5%) | 184.4 (30.4%) | <0.001 |
| Watch category (%) | 429.3 (68.7%) | 1,395.5 (92.6%) | 409.8 (67.6%) |  |
| Reserve category (%) | 12.4 (2.0%) | 25.1 (1.9%) | 12.1 (2.0%) |  |

*per patients **per admissions ***Severe infection is defined by four consecutive days of parenteral antibiotics, or died, were discharged to a hospice or transferred to other hospital before completing four consecutive days of antibiotics and had antibiotics continuously until the day prior to death, a hospice discharge or transfer
