# Supplementary material for: Diagnostic and antibiotic use practices among COVID-19 and non-COVID-19 patients in the Indonesian National Referral Hospital
Source: PLoS One. 2024 Mar 7;19(3):e0297405. doi: 10.1371/journal.pone.0297405 (PMC10919621; doi:10.1371/journal.pone.0297405)
Supplement: S2 Table — (DOCX) [file pone.0297405.s008.docx]

**S2 Table.** Mortality, proportion of having blood culture sampled and median time to parenteral antibiotics discontinuation of patients with severe infection within 28 days after parenteral antibiotics being started, stratified by variables and COVID-19 status

| **Variables** | **28-day mortality (%) ^a^** | | **Proportion of having blood culture sampled (%)** | | **Median time to**  **discontinuation of parenteral antibiotics (IQR)** | |
| --- | --- | --- | --- | --- | --- | --- |
|  | **COVID-19** | **Non-COVID-19** | **COVID-19** | **Non-COVID-19** | **COVID-19** | **Non-COVID-19** |
| **Sex** |  |  |  |  |  |  |
| Female | 37/156 (23.7%) | 2231/9445 (23.6%) | 64/156 (41.0%) | 2941/9445 (31.1%) | 6 (5-11) | 5 (5-9) |
| Male | 58/177 (32.8%) | 2254/9392 (24.0%) | 85/177 (48.0%) | 3313/9392 (35.3%) | 6 (5-12) | 6 (5-10) |
| **Age (years old)** |  |  |  |  |  |  |
| <1 | 0/6 (0%) | 380/1844 (20.6%) | 4/6 (66.7%) | 1479/1844 (80.2%) | 7 (5-28) | 8 (5-18) |
| 1-4 | 6/19 (31.6%) | 255/1332 (19.1%) | 16/19 (84.2%) | 771/1332 (57.9%) | 5 (5-8) | 6 (5-11) |
| 5-14 | 9/29 (31.0%) | 281/1455 (19.3%) | 26/29 (89.7%) | 758/1455 (52.1%) | 7 (5-14) | 5 (5-9) |
| 15-24 | 5/25 (20.0%) | 386/1616 (23.9%) | 19/25 (76.0%) | 463/1616 (28.7%) | 7 (5-20) | 5 (5-8) |
| 25-34 | 6/38 (15.9%) | 465/2057 (22.6%) | 11/38 (28.9%) | 339/2057 (16.5%) | 5 (4-8) | 5 (5-7) |
| 35-44 | 8/38 (21.1%) | 607/2486 (24.4%) | 11/38 (28.9%) | 506/2486 (20.4%) | 5 (4-8) | 5 (5-8) |
| 45-54 | 12/50 (24%) | 698/2787 (25.0%) | 22/50 (44.0%) | 672/2787 (24.1%) | 7 (5-13) | 5 (5-9) |
| 55-64 | 18/63 (28.6%) | 731/2866 (25.5%) | 16/63 (25.4%) | 707/2866 (24.7%) | 6 (4-11) | 6 (5-10) |
| > 65 | 31/65 (47.7%) | 682/2394 (28.5%) | 24/65 (36.9%) | 559/2394 (23.4%) | 6 (5-13) | 6 (5-10) |
| **Admission year** |  |  |  |  |  |  |
| 2019 | - | 2282/9433 (24.2%) | - | 3113/9433 (33.0%) | - | 5 (5-10) |
| 2020 | 95/333 (28.5%) | 2203/9404 (23.4%) | 149/333 (44.7%) | 3141/9404 (33.4%) | 6 (5-11) | 5 (5-9) |

**^a^** Mortality included in-hospital mortality and discharged to a hospice.
